# Perioperative incidence of ECMO and IABP on 5901 mitral valve surgery procedures

**DOI:** 10.1186/s13019-022-01790-1

**Published:** 2022-03-17

**Authors:** Ignazio Condello, Roberto Lorusso, Giuseppe Santarpino, Nicola Di Bari, Flavio Fiore, Marco Moscarelli, Antonio Maria Calafiore, Giuseppe Speziale, Giuseppe Nasso

**Affiliations:** 1 Department of Cardiac Surgery, Perfusion Service, Anthea Hospital, GVM Care and Research, Via Camillo Rosalba 35/37, 70124 Bari, Italy; 2 Cardio-Thoracic Surgery Department, Heart and Vascular Centre, Maastricht University Medical Centre, Maastricht, The Netherlands; 3 Cardiovascular Research Institute Maastricht, Maastricht, The Netherlands; 4 Department of Cardiac Surgery, Paracelsus Medical University, Nuremberg, Germany; 5 Cardiac Surgery Unit, Department of Experimental and Clinical Medicine, “Magna Graecia” University of Catanzaro, Catanzaro, Italy; 6 Division of Cardiac Surgery, Dipartimento Di Emergenza E Trapianti Di Organo (D.E.T.O.), University of Bari, Bari, Italy

**Keywords:** Extracorporeal membrane oxygenation, Intra-aortic balloon pump, Low cardiac output syndrome, Postcardiotomy cardiogenic shock, Right mini-thoracotomy, Full sternotomy, Mitral valve surgery

## Abstract

**Background:**

Report the incidence and results of peri-operative extracorporeal membrane oxygenation (ECMO) and intra-aortic balloon pump (IABP) of patients undergoing mitral valve surgery (MVS) through right mini-thoracotomy (RT) and conventional full sternotomy (FS) for a period of 6 years from eleven tertiary Cardiac Surgery Institutes of GVM Care & Research Italia.

**Methods:**

From January 2016 to November 2021, a total of 5901 consecutive patients underwent MVS through RT and FS. The primary outcome of the study was the mortality and incidence of low cardiac output syndrome (LCOS) treated with intra-aortic balloon pump (IABP) with or without inotropic support and the incidence of Postcardiotomy Cardiogenic Shock (PCS) treated with Veno-arterial (VA) Extracorporeal Membrane Oxygenation (ECMO) on patients undergoing mitral valve surgery (MVS) through right mini-thoracotomy (RT) versus conventional full sternotomy (FS).

**Results:**

The mean age was 66 ± 15 years, 3389 patients underwent in RT approach 2512 in FS, 3081 (52%) patients were male and 2.3% had previous cardiac operations. Cardiopulmonary bypass time was 93 min for RT and 81 min for FS and cross clamp time 75 min for RT and 63 min for FS for mitral valve repair. Incidence of perioperative IABP for the treatment of low cardiac output was reported on 99 patients (1.6%), 51 for RT (1.5%), 35% used inotropic support (adrenaline and milrinone) and 48 in FS (1.9), 28% use inotropic support, 21 patients died after IABP (3 RT and 18 FS). Incidence of perioperative VA-ECMO for the PCS treatment was 13 and 4 with IABP, 9 RT (0.2%) and 4 FS approach (0.15%), 12 patients died after VA-ECMO.

**Conclusion:**

Minimally invasive mitral valve surgery is a safe and reproducible approach associated with low mortality and morbidity. ECMO and IABP incidence for the treatment of PCS was 0.2% and for Low cardiac output syndrome (LCOS) was 1.6% in elective mitral valve surgery is very low. The patients that use the perioperative IABP in minimally invasive mitral valve surgery (MIMVS) trough RT reported a reduced mortality compared to FS in relation to the operative risk and surgical technique. Low incidence of VA-ECMO was found in RT and FS approach, only one patient survived after VA-ECMO after minimally invasive mitral valve surgery.

## Background

Mitral valve surgery using conventional full sternotomy (FS) is the conventional approach for the treatment of the mitral valve disease. Despite this procedure has shown excellent postoperative outcomes, in the last two decades minimally invasive mitral valve surgery (MIMVS) has gained consensus among surgeons as it has provided greater patients satisfaction, maintaining the same quality and safety of the standard mitral valve surgery approach [1, 2]. Intra-aortic balloon pump (IABP) is the most usable tool of temporary mechanical circulatory support for cardiac surgical patients suffered from low cardiac output in the early postoperative phase. Its beneficial action is attributed to a concomitant reduction in afterload of left ventricle with a substantial increase on coronary perfusion pressure due to an increase of aortic diastolic pressure. The main indication of IABP use in cardiac surgical patients is peri and post-operatively in the treatment of a low cardiac output state refractory to the usual inotropic support. In literature the 30-day mortality for the patients necessitating IABP is high because of the cardiac problems that led to the need for this pump, ranged from 26 to 50% [3]. Cardiogenic shock, cardiac arrest, acute respiratory failure, or a combination of such events, are all potential complications after cardiac surgery which lead to high mortality [4]. Use of extracorporeal temporary cardio-circulatory and respiratory support for progressive clinical deterioration can facilitate bridging the patient to recovery or to more durable support. Over the last decade, extracorporeal membrane oxygenation (ECMO) has emerged as the preferred temporary artificial support system in such circumstances [5]. Aim of this study was to analyze the incidence and results of perioperative extracorporeal membrane oxygenation (ECMO) and intra-aortic balloon pump (IABP) of patients undergoing mitral valve surgery (MVS) through right mini-thoracotomy (RT) and conventional full sternotomy (FS) for a period of 6 years of eleven Cardiac Surgery Institutes of GVM Care & Research Italia.

## Methods

### Patient and data collection

A retrospective, observational study was undertaken of prospectively collected data in 5901 consecutive patients undergoing mitral valve surgery, of which 3389 underwent MIMVS through RT between January 2016 to November 2021 for a period of 6 years from 11 Cardiac Surgery Institutes of GVM Care & Research Italia. Two-five hundred twelve procedures were performed in sternotomy. The main reason for performing a sternotomy approach was the selection patient for the learning curve, and in case of very poor left ventricular ejection fraction, strong pleural adhesions, severe chronic obstructive pulmonary disease and active endocarditis with abscess involving the mitro-aortic continuity. All patients signed an informed consent form to allow clinical and administrative data storage and utilization for scientific purposes according to the General Data Protection Regulation. Because of the retrospective nature of this study, the local ethics committees waived the need for patient consent. The primary outcome of the study was the mortality and incidence of low cardiac output treated with intra-aortic balloon pump (IABP) with or without inotropic support and the incidence of Postcardiotomy Cardiogenic Shock (PCS) treated with Veno-arterial (VA) extracorporeal membrane oxygenation (ECMO) on patients undergoing mitral valve surgery (MVS) through right mini-thoracotomy (RT) vs conventional full sternotomy (FS).

Low cardiac output was defined as the need for postoperative inotropic support for more than 48 h in the intensive care unit and/or from an intra-aortic balloon pump.

### Surgical technique

The standardized surgical approach for MIMVS has been reported elsewhere. Briefly, MIMVS by a way of right anterior thoracotomy was performed through a 5–7 cm skin incision placed at 3th or 4th intercostal space. Two trocars are inserted in the thorax to allow positioning of a ventricular vent, CO_2_ insufflator, camera device and pericardial stay sutures. Whereas at the beginning of our experience the approach involved retrograde arterial perfusion and balloon endoclamping, the procedure has evolved to a technique with ascending aorta cannulation, long femoral venous cannula drainage, and direct transthoracic aortic clamping. Specifically, direct aortic cannulation was performed using Easyflow (Sorin, Salluggia, Italy) or Straightshot (Edwards Lifesciences, Irvine, Calif) cannulas. Biomedicus single stage (Medtronic, Minneapolis, Minn) or RAP single 2 stage cannulas (Estech) were inserted through the femoral vein into the right atrium and the correct position was achieved with the Seldinger technique under transesophageal echocardiographic guidance. In case of mitral and tricuspid valve surgery, a single 2 stage cannula (RAP, Estech) was used as it allows to drain simultaneously the superior and inferior venae cavae. After vacuum-assisted cardiopulmonary bypass (− 40 to − 60 mmHg) was established, the patients were cooled to 34 °C. the ascending aorta was clamped with the Cygnet crossclamp (ovare surgical System, Cupertino, Calif) or with the aortic clamp (Cardiomedical GmbH, Langenhagen, Germany; distributed by sorin, Salluggia, Italy) and antegrade cold crystalloid or warm blood cardioplegia is delivered directly into the ascending aorta by a needle vent catheter. For conventional approach on mitral valve surgery was performed in median sternotomy with central cannulation [6].

The mitral valve is approached with a traditional left paraseptal atriotomy and exposed using a specially designed atrial retractor held by a mechanical harm inserted through a right parasternal port. Mitral valve procedures were performed under a combination of direct vision and thoracoscopic assistance. All patients received an accurate intraoperative transoesophageal echocardiogram before and after weaning from cardiopulmonary bypass machine. In patients who had an attempt to repair, our policy is to replace the mitral valve if (a) at the hydrostatic saline test after several attempts, there is still some degrees of mitral regurgitation, (b) the surface of coaptation is not enough to guarantee a long durability, (c) at intraoperative echo, there is more than mild mitral regurgitation. Twelve surgeons contributed to this series, with 2 of them (GS, GN) performing 45.2% of the operations.

### IABP indication and management

The indications for initiating treatment with IABP in this patients was the following: (a) IABP support for persistent preoperative ischemia despite maximum medical treatment (b) patients not able to be discontinued from CPB although forced inotropic support, (c) patients in low-cardiac output status just after a "difficult" discontinuation of CPB, supported by high-doses of inotropes, (d) patients with "difficult" discontinuation from CPB and spontaneous appearance of arrhythmia (premature ventricular beats or VT) not amenable in anti-arrhythmic continuous infusion and (e) post cardiotomy low cardiac output syndrome. Prophylactic initiation of IABP treatment was not advocated in any of the cases [7, 8]. A Datascope system (Datascope Corp, Paramus, NJ) was utilised. The IABP was introduced percutaneously through the common femoral artery in 95 patients and through an open access of the femoral artery in the remaining 4 patients [9]. Correct placement of the device was routinely confirmed with Chest X Ray in ICU. Once mediastinal drainage was minimum (< 50 ml/h), patients were anticoagulated with Heparin infusion, keeping the ACT > 180–200 s [10, 11].

### ECMO indication and management

ECMO was initiated intraoperatively in the operating room for circulatory instability during or immediately after weaning from the cardiopulmonary bypass (CPB) in the primary cardiac procedure. The capability to institute ECMO secondarily in the ICU, for delayed PCS or cardiac arrest, was available. The clinical criteria for PCS included the following: left atrial pressure > 15 mmHg; central venous pressure > 12 mmHg; metabolic acidosis (i.e. pH < 7.3 with serum lactate > 3.0 mmol/L); end-organ hypoperfusion (urine output < 30 mL/h); cardiac index < 2.2 L/min/m^2^; and systolic blood pressure < 80 mmHg despite adequate filling volumes, use of multiple adrenergic agents (epinephrine > 0.1 µg/kg/min or dobutamine > 10 µg/kg/min, norepinephrine > 0.1 µg/kg/min), or an intra-aortic balloon pump (IABP). VA-ECMO support was initiated via peripheral cannulation through the femoral route with the semi-open method, and an additional 6 Fr catheter was systematically inserted distally into the femoral artery to prevent leg ischemia [12]. ECMO blood flow was adjusted on based on clinical assessments (e.g. pre-oxygenator venous oxygen saturation, evidence of hypoperfusion, resolution of hyperlactatemia, normalization of mean arterial pressure). Intravenous unfractionated heparin was given to maintain an activated clotting time of 180–210 s, or an activated partial thromboplastin time of 1.5–2 times normal. ECMO-related complications were carefully monitored. ECMO weaning was performed in patients who fulfilled our published institutional weaning criteria and passed an ECMO weaning trial consisting in decreasing and clamping ECMO flow. In general, the patient should have a pulsatile arterial waveform for at least 24 h; be hemodynamically stable, with baseline mean arterial pressure greater than 60 mmHg with no or low doses of catecholamines; should have left ventricular ejection fraction (LVEF) of 35%, and an aortic velocity time integral (VTI) of ≥ 12 cm; and have recovered from major metabolic disturbances [13]. Weaning was considered unsuccessful if ECMO re-cannulation was required within 2 days of decannulation.

### Statistical analysis

Continuous data were expressed as mean ± standard deviation or median with the interquartile range and categorical data as percentages. All statistical analyses were performed with SPSS 22.0 (SPSS, Inc., Chicago, IL, USA).

## Results

The mean age was 66 ± 15 years, 3389 patients underwent in RT approach 2512 in FS, 3081 (52%) patients were male and 2.3 had previous cardiac operations, the patient characteristics were reported in (Table 1).

**Table 1:** Characteristics of patients for MIMVS and FS

	All	MIMVS	FS	*P* value
	*n* = 5901	*n* = 3389	*n* = 2512	
Male gender	52.5%	45.9%	39.9%	< 0.001
Age (years)	66 (62–76)	65 (62–78)	69 (67–79)	< 0.001
Body mass index (kg/m^2^)	25.8 (22.2–28.8)	26.8 (23.4–29.0)	24.3 (21.7–28.4)	0.191
Arterial hypertension	36.2%	35.3%	36.1%	0.789
Diabetes mellitus				
Oral antidiabetic drugs	8.9%	7.2%	5.5%	0.006
Insulin	2.3%	2.8%	2.6%	0.530
Hypercholesterolaemia	41.2%	42.1%	44.3%	0.009
Renal dysfuntion	1.4%	2.3%	2.1%	0.254
Respiratory or lung disease	2.1%	2.3%	1.9%	0.187
Previous disabling stroke	1.5%	1.5%	1.3%	0.687
History of cancer	1.4%	1.9%	2.5%	0.030
Atrial fibrillation	9.4%	8.3%	11.2%	< 0.001
Peripheral vascular disease	4.8%	4.3%	4.9%	0.556
Coronary artery disease	9.3%	10.2%	8.4%	< 0.001
Left ventricular ejection fraction				0.347
LVEF > 50%	93.3%	92.4%	91.4%	
LVEF 30–50%	6.0%	7.1%	7.3%	
LVEF < 30%	0.7%	0.5%	1.3%	
Previous surgery	2.3%	1.7%	1.9%	0.211
EuroSCORE II (%)	1.2 (1.1–2.8)	1.4(1.1–2.7)	1.9 (1.3–3.1)	0.387
Urgent procedure	2.6%	2.8%	3.1%	0.338

Median (interquartile range) or percentage. Renal dysfunction: dialysis or creatinine > 2 mg/dl

LVEF, left ventricular ejection fraction; MIMVS, minimally invasive mitral valve surgery; FS, full sternotomy

Cardiopulmonary bypass time was, 93 min for RT vs 81 min for FS and cross clamp time, 75 min for RT vs 63 min for FS for mitral valve repair (Table 2). The most predominant pathology was degenerative disease, followed by functional mitral valve regurgitation rheumatic disease, endocarditis and prosthetic dysfunction (Tables 3, 4). Mitral valve repair was performed in 3207 patients 78% in RT and 2694 had mitral valve replacement 58% in RT. Direct aortic cannulation was achieved in 825 patients. Repair techniques included annuloplasty, leafleat resection, neochordae implantation and sliding plasty (11%). Concomitant procedures included tricuspid valve repair (14.6%), atrial fibrillation ablation (9.5%) and atrial septal defect closure (3.2%). Overall in-hospital mortality was 1.1%. 78 had conversion to sternotomy. Incidence of perioperative IABP for the treatment of LCOS was reported on 99 patients (1.6%), 51 (1.5%) for RT, 35% used inotropic support (adrenaline and milrinone) and 48 in FS (1.9%), 28% use inotropic support, 21 patients died after IABP (3 RT vs 18 FS) (Tables 5, 6). Incidence of perioperative VA-ECMO for the PCS treatment was 13 (0.2%) and 4 with IABP, (9 RT vs 4 FS approach), 12 patients died after VA-ECMO (Table 7).

**Table 2:** Intraoperative data for surgical techniques and procedures

Surgical approach	RT	FS
	*n* = 491	*n* = 513
*Valve replacement* n. tot = 1004		
Cardiopulmonary bypass time (min)	82 (79–98)	84 (80–99)
Cross-clamp time (min)	59 (43–69)	62 (45–71)

	*n* = 2898	*n* = 1999
*Valve repair* n. tot = 4897		
Cardiopulmonary bypass time (min)	93 (62–99)	81 (54–89)
Cross-clamp time (min)	75 (37–76)	63 (36–73)

RT, right thoracotomy; FS, full sternotomy

**Table 3:** Mitral valve pathology for minimally invasive mitral valve surgery

Mitral valve pathology	Right-thoracotomy	Repair	Replacement
	*N* = 3389	*N* = 2898	*N* = 491
Degenerative	2739 (80.8%)	2540 (87.6%)	199 (40.5%)
Functional	328 (9.6%)	231 (7.9%)	97 (19.7%)
Reumathic	247 (7.2%)	97 (3.3%)	150 (30.5%)
Endocarditis	28 (0.8%)	3 (1.0%)	25 (5.0%)
Prosthesis dysfunction	19 (0.5%)	1 (0.03%)	18 (3.6%)
Miscellaneous	28 (0.9%)	26 (0.9%)	2 (0.4%)

**Table 4:** Mitral valve pathology for full sternotomy mitral valve surgery

Mitral valve pathology	Full Sternotomy	Repair	Replacement
	*N* = 2512	*N* = 1999	*N* = 513
Degenerative	1781 (70.8%)	1639 (81.9%)	142 (27.6%)
Functional	367 (14.6%)	179 (8.9%)	188 (36.6%)
Reumathic	289 (11.5%)	143 (6.6%)	146 (28.4%)
Endocarditis	29 (1.15%)	7 (0.3%)	22 (4.2%)
Prosthesis dysfunction	17 (0.67%)	10 (0.5%)	7 (1.36%)
Miscellaneous	29 (1.15%)	21 (1.0%)	8 (1.5%)

**Table 5:** Perioperative incidence of IABP for Mitral valve pathology in Full Sternotomy (FS)

IABP	FS	Repair	Replacement	Inotropic support	Death 30 days
	*N* = 48	*N* = 26	*N* = 22	28%	18
Degenerative		15	1		
Functional			11		4 Repair and 5 Replacement
Reumathic			4		1 Replacement
Endocarditis		5	3		3 Repair
Prosthesis dysfunction		6	3		2 Replacement and 3 Repair
Miscellaneous					

IABP, intra-aortic balloon pump; FS: full sternotomy

**Table 6:** Perioperative incidence of IABP for mitral valve pathology in MIMVS

IABP	RT	Repair	Replacement	Inotropic support	Death 30 days
	*N* = 51	*N* = 22	*N* = 29	35%	3
Degenerative					
Functional		13	18		
Reumathic			1		1 Replacement
Endocarditis		4	1		
Prosthesis dysfunction		5	9		2 Replacement
Miscellaneous					

IABP, intra-aortic balloon pump; MIMVS, minimally invasive mitral valve surgery; RT, right thoracotomy

**Table 7:** Perioperative incidence and mortality of ECMO in FS and RT approach

	VA-ECMO	IABP	Inotropic support	Survival
FS	4	1		
RT	9	3		1
Tot	13	4	53%	

IABP, intra-aortic balloon pump; RT, right thoracotomy; FS, full sternotomy VA-ECMO, Veno-arterial extracorporeal membrane oxygenation

## Discussion

Despite this study reports a good result for mitral valve surgery both in FS and MIMVS, the perioperative LCOS the most serious complication and is associated with increased morbidity, short- and long-term mortality, and healthcare resource utilization.

The presented analysis represents a pilot of our experience. Due to these preliminary results we are going to prepare a statistical analysis with a comparison between two comparable groups and multivariate. This syndrome is characterized by decreased heart pump function, leading to reduced oxygen delivery (DO_2_) and subsequent tissue hypoxia. The most common definition of LCOS also includes decreases in the cardiac index (CI) to < 2.0 L/min/m^2^ and a systolic blood pressure of < 90 mmHg, in conjunction with signs of tissue hypoperfusion (cold periphery, clammy skin, confusion, oliguria, elevated lactate level) in the absence of hypovolemia. The use of inotropic agents or mechanical circulatory support always is required to improve patient hemodynamics. Although studies have reported that the occurrence of LCOS may be related to preoperative cardiac function, intraoperative operations and CPB, there are still no exact indicators to reflect the risk of its occurrence. At present, domestic and foreign studies on the risk factors of LCOS are inconsistent [14]. It is believed that the occurrence of LCOS is caused by multiple factors, including impaired systolic and diastolic function of the heart, changes in cardiac load, and activation of inflammatory transmitters [15]. The patients that use the perioperative IABP in minimally invasive mitral valve surgery (MIMVS) trough RT report a reduced mortality compared to FS in relation to the operative risk and surgical technique, this could be related to the elimination of sternotomy, which reduces the time of mechanical ventilation and the postoperative recovery time [6].

Postcardiotomy cardiogenic shock (PCS), defined as low cardiac output syndrome with evidence of tissue hypoperfusion and end-organ dysfunction despite adequate preload, affects 0.2% to 6% of patients who undergo cardiothoracic surgery [15]. PCS is a life-threatening complication with mortality rates between 50 and 80% and includes the inability to wean from cardiopulmonary bypass (CPB) in the operating room or deterioration of myocardial function during the first postoperative days. Between 70 and 90% of the patients who cannot be easily separated from CPB because of PCS can be weaned from CPB by support of inotropes, vasopressors, and intra-aortic balloon pumps, and an estimated two thirds of these patients will recover hemodynamically without the need for other mechanical circulatory support. In comparison, PCS refractory to intravascular volume loading, pharmacologic, and intra-aortic balloon pump support occurs postoperatively in 0.5–1.5% of patients and will inexorably lead to death unless more efficient circulatory support is initiated [16]. The main limitation of this study is its retrospective nature; however our database is prospectively compiled. Although we reported the incidence of LOCS and PCS results, echocardiographic data were not available for all patients. No information has been recorded regarding the aetiology of perioperative myocardial infarction as well as no information has been reported regarding the cause of late mortality. Nevertheless a well-designed study with appropriate sample size is required to validate this results.

## Conclusion

ECMO and IABP incidence for the treatment of PCS was 0.2% and for Low cardiac output syndrome (LCOS) was 1.6% in elective mitral valve surgery is very low in right mini-thoracotomy (RT) and conventional full sternotomy (FS). The patients that use the perioperative IABP in minimally invasive mitral valve surgery (MIMVS) trough RT reported a reduced mortality compared to FS in relation to the operative risk and surgical technique. Only one patient survived after VA-ECMO after minimally invasive.

## Data Availability

The datasets used and analysed during the current study are available from the corresponding author on reasonable request.

## Abbreviations

MIMVS: Minimally invasive mitral valve surgery
FS: Full sternotomy
RT: Right thoracotomy
MVS: Mitral valve surgery
IABP: Intra-aortic balloon pump
VA-ECMO: Veno-arterial extracorporeal membrane oxygenation
LCOS: Low cardiac output syndrome
PCS: Post cardiotomy shock
